# Supplementary material for: Ultrafast adaptive optics for imaging the living human eye
Source: Nat Commun. 2024 Nov 29;15:10409. doi: 10.1038/s41467-024-54687-z (PMC11607088; doi:10.1038/s41467-024-54687-z)
Supplement: Supplementary file 1 — Supplementary Information [file 41467_2024_54687_MOESM1_ESM.pdf]

Supplementary Information

Ultrafast adaptive optics for imaging the living human eye

Yan Liu[1,*], James A. Crowell[1], Kazuhiro Kurokawa[1,2,†], Marcel T. Bernucci[1,†], Qiuzhi Ji[1,†], Ayoub Lassoued[1,3], Hae Won Jung[1,4], Matthew J. Keller[1], Mary E. Marte[1,5], Donald T. Miller[1,*]

[1]School of Optometry, Indiana University, Bloomington, Indiana, United States.
[2]Current affiliation: Discoveries in Sight Research Laboratories, Devers Eye Institute, Legacy Research Institute, Legacy Health, Portland, Oregon, United States.
[3]Current affiliation: Centre Hospitalier National d'Ophtalmologie des Quinze-Vingts Centre d'investigation clinique, Paris, Île-de-France, France; Institut de la vision, Paris, Île-de-France, France.
[4]Current affiliation: University of Houston, Houston, Texas, United States.
[5]Current affiliation: Richard L. Roudebush VAMC, Indianapolis, Indiana, United States.
[†]These authors contributed equally to this work
[*]Email: yl144@iu.edu, dtmiller@iu.edu

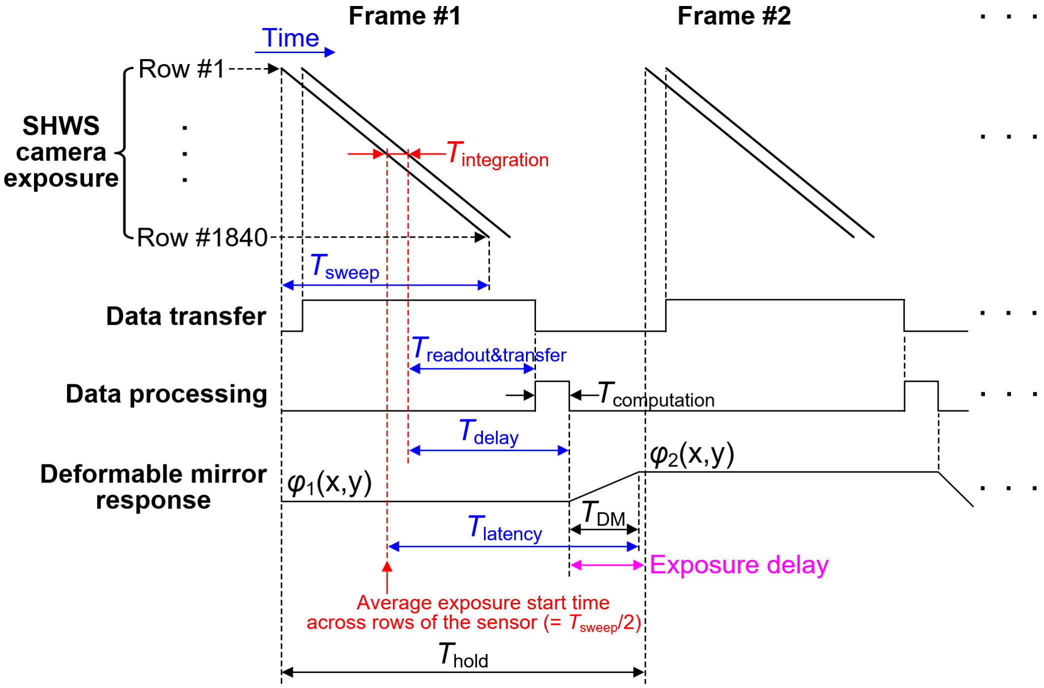

Supplementary Figure 1. Timing diagram for our ultrafast AO using the discontinuous-exposure scheme. The Shack-Hartmann wavefront sensor (SHWS) camera (ORCA-Lightning, Hamamatsu) uses a rolling shutter that sweeps from top to bottom of the sensor; it takes T_{sweep} = 2.9 ms to sweep through the 1840 rows that we use for wavefront sensing. Because the AO system modeling presented in Supplementary Note 2 assumes a global shutter that exposes all rows simultaneously, we convert our rolling shutter exposure process to the equivalent global shutter exposure process [1, 2]. A comparison between the rolling shutter and global shutter modes for the SHWS camera in AO ophthalmoscopy is presented in Supplementary Note 1. In this equivalent global exposure mode, all rows start their exposure at $T_{sweep}/2$ = 1.45 ms (i.e. the average exposure start time across rows of the camera, labeled in the figure) and expose for $T_{integration}$ = 0.126 ms long. In addition, the time for pixel readout and data transfer $T_{readout\&transfer}$ = 1.93 ms; the time for data processing on a computer $T_{computation}$ = 0.49 ms; the time delay due to pixel readout, data transfer and data processing $T_{delay} = T_{readout\&transfer} + T_{computation}$ = 2.42 ms; the period for the deformable mirror (DM) to hold a wavefront correction pattern T_{hold} = 1/233 Hz = 4.3 ms; the nominal response time of the DM $T_{DM} \approx 0.55$ ms (see Supplementary Note 2). To minimize the impact of DM actuation, we introduced a delay (labeled as "Exposure delay") between sending control commands to the DM and the start of the next SHWS exposure. We vary the exposure delay in Supplementary Note 4 to investigate how DM actuation affects AO performance and to determine the optimal delay for eliminating the DM actuation effect while maximizing AO bandwidth.

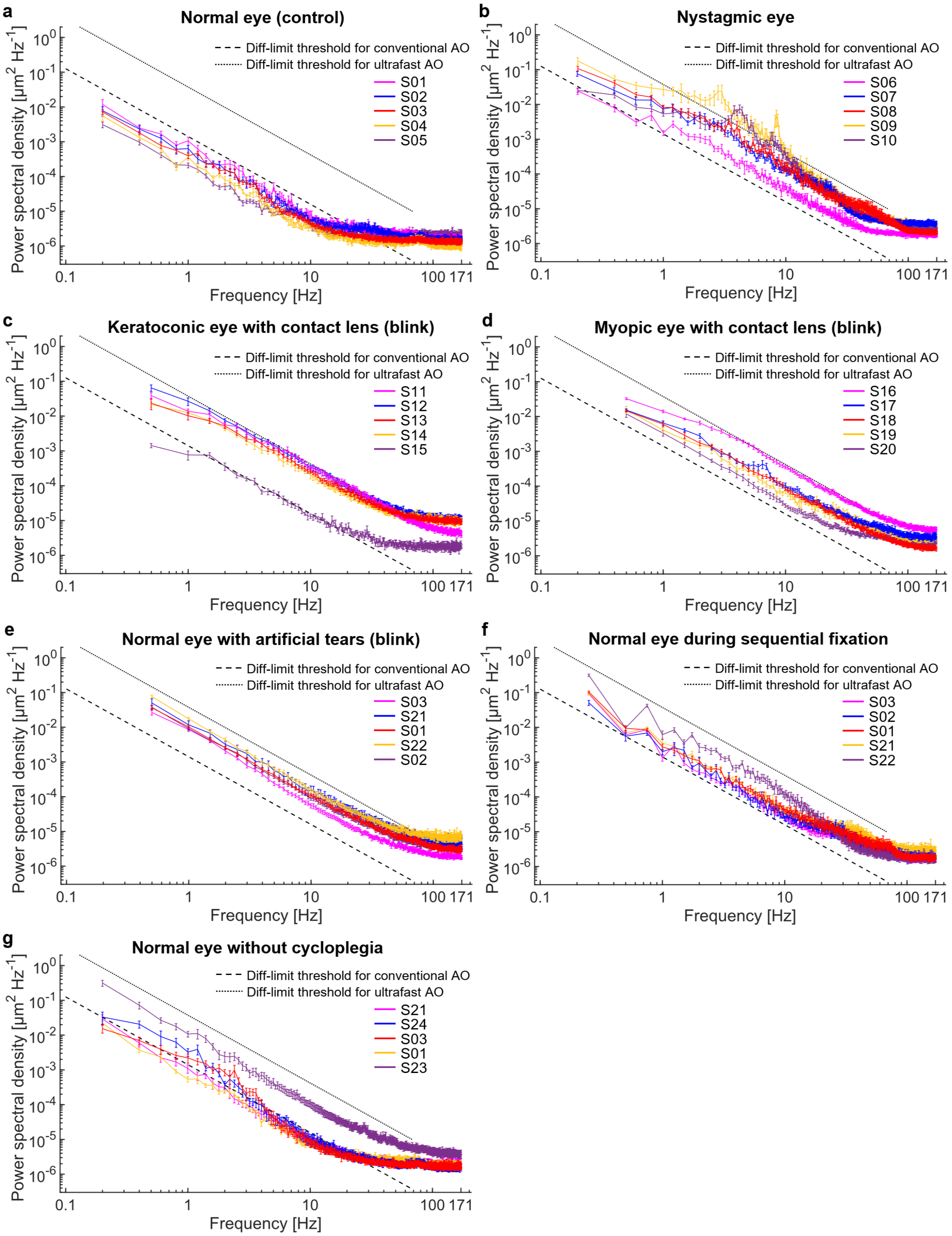

Supplementary Figure 2. Power spectra of ocular aberrations for each subject in each scenario, co-plotted with diffraction-limit (diff-limit) threshold for conventional and ultrafast AO. Five subjects were measured for each scenario. The red curve within each scenario corresponds to the median subject of the five subjects measured, and the associated imaging and AO performance are shown in Figures 5−9 of the main text. All power spectra measurements were based on 5-second long videos, except for 1) the blink scenarios where we analyzed the 2-second long data acquired after eye reopened from blinks and 2) the sequential fixation

scenario where we analyzed the 4-second long video. Error bars denote standard errors (15 repeated measurements for each nystagmic eye, 10 repeated measurements for each eye in all other scenarios). Diffraction-limit thresholds are defined in the main text. Subject information is given in Supplementary Table S1. Note that Subject S15 has a small pupil size (4 mm), which resulted in a lower power spectrum than those of other four keratoconus subjects in Supplementary Figure S2c. As such, this subject data was excluded when we calculated the mean power spectrum for the keratoconic eye scenario shown in Figure 4a. Also note that 27 out of 30 power spectra in clinically-relevant scenarios exceed the diffraction-limit threshold for conventional AO, highlighting the need for higher AO speeds than that provided by conventional AO.

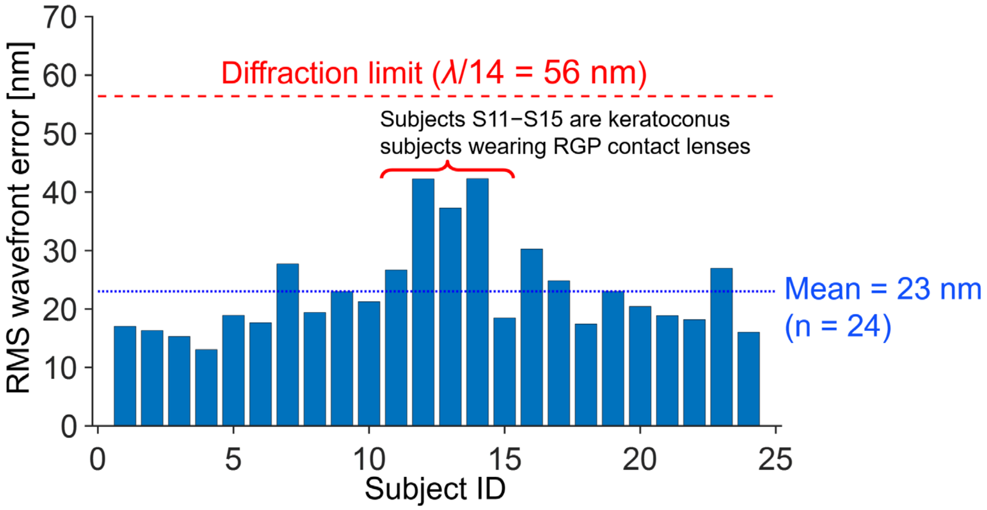

Supplementary Figure 3. RMS wavefront error attributed to noise was estimated for each of the 24 subjects (S01 to S24) using their power spectra data shown in Supplementary Figure 2. Subject S25 has left the country so that we could not measure his aberration power spectrum. The RMS wavefront errors of S11 to S14 (keratoconus eyes wearing rigid gas permeable (RGP) contact lenses) are higher than normal, may be because the RGP lenses cause more scattering/reflections at 790 nm.

Supplementary Table 1 Subject information

Subject ID	Internal subject ID #	Age (y/o)	Sex	Scenario participated *	Eye imaged	Eye condition
S01	286	23	F	1, 2, 3, 5	OD	Normal
S02	276	22	F	1, 2, 5	OD	Normal
S03	277	31	M	1, 2, 3, 5	OD	Normal
S04	154	31	M	1, 4	OD	Normal
S05	198	31	M	1	OD	Normal
S06	250	57	M	8	OD	Nystagmus
S07	246	48	F	8	OD	Nystagmus
S08	187	26	M	8	OD	Nystagmus
S09	274	45	F	8	OD	Nystagmus
S10	245	30	M	8	OD	Nystagmus
S11	289	63	F	6	OS	Keratoconus. RGP lens: Sph = −12 D, Art Optical AKS
S12	253	57	M	6	OD	Keratoconus. RGP lens: Sph = +3.00 D, Cyl = −2.75 D, Ax = 100, Blanchard RoseK2
S13	244	64	M	6	OD	Keratoconus. RGP lens: Sph = −5.25 D, Art Optical RoseK2
S14	249	65	F	6	OD	Keratoconus. RGP lens: Sph = −2.00 D, Blanchard RoseK2
S15	248	76	M	6	OS	Keratoconus. RGP lens: Sph = −2.00 D, Blanchard RoseK PG
S16	242	27	M	7	OD	High myopia. Soft lens: Sph = −10 D, Cyl= −1.25 D, Ax = 160
S17	278	23	F	7	OD	High myopia. Soft lens: Sph = −6 D, Cyl = −1.25 D, Ax = 180
S18	195	27	F	7	OD	High myopia. Soft lens: Sph = −6.5 D
S19	241	22	F	7	OD	High myopia. Soft lens: Sph = −6 D
S20	280	29	M	7	OD	High myopia. Soft lens: Sph = −8 D, Cyl = −0.75 D, Ax = 180
S21	285	20	F	2, 3, 5	OD	Normal
S22	053	56	M	2, 5	OD	Normal
S23	196	25	M	3	OD	Normal
S24	204	24	F	3	OD	Normal
S25	116	30	M	3	OD	Normal

*ID for different experimental scenarios:

1. Normal eye (control)
2. Normal eye with artificial tears (blink)
3. Normal eye without cycloplegia
4. Normal eye without cycloplegia (blink)
5. Normal eye with sequential fixation
6. Keratoconic eye with an RGP (Rigid Gas Permeable) contact lens (blink)
7. Myopic eye with a soft contact lens (blink)
8. Nystagmic eye

Supplementary Note 1: Comparing rolling shutter and global shutter modes for the SHWS camera in AO ophthalmoscopy

Rolling shutter and global shutter cameras use fundamentally different exposure schemes for capturing images. In global shutter mode, all pixels begin exposing at the same time, capturing the entire image at once. By contrast in rolling shutter mode, adjacent rows of pixels begin exposing at slightly different times, capturing the image line by line. This staggered exposure can lead to distortion in the image for objects or scenes that are moving faster than the rolling shutter speed.

A key advantage of global shutter cameras is that they do not suffer from such image distortion. However, they have a number of disadvantages compared to rolling shutter cameras that may outweigh this advantage.

1. **Lower frame rate:** Global shutter cameras require a reference frame to be read out from the sensor in addition to the signal frame. As a result, the maximum frame rate of global shutter is only half that of rolling shutter cameras [3]. This lower frame rate leads to a reduced AO loop rate, which reduces AO bandwidth and power rejection performance.

2. **Increased noise:** Global shutter cameras generally exhibit higher noise levels. Their RMS readout noise is at least 1.41 times greater than that of rolling shutter cameras, due to the additional frame mentioned in (1). This results in lower sensitivity.

3. **Lower quantum efficiency:** Global shutter cameras often have lower quantum efficiency because their pixel architecture includes extra electronics for the shuttering mechanism, which reduces the effective area available for light collection and results in a lower fill factor. Note that the quantum efficiency of a camera includes non-material related loss factors such as the fill factor [4].

4. **Smaller market:** The market for global shutter cameras is smaller with fewer options available. This is particularly true for scientific CMOS (sCMOS) cameras, which are attractive for wavefront sensing in the eye because of their high sensitivity, speed, dynamic range and field of view. For example, sCMOS cameras sold by Hamamatsu (the manufacturer of the SHWS camera used in our study) are only available with rolling shutters.

5. **Higher cost:** Global shutter cameras are generally more expensive due to their more complex pixel architecture and circuitry, as well as their smaller market size.

Which shutter mode is better depends on the specific application. For AO ophthalmoscopy, rolling shutter mode allows up to twice the AO loop rate compared to global shutter mode, improving power rejection performance and increasing AO bandwidth. In addition, the reduced noise and higher quantum efficiency of rolling shutter mode improve the centroiding accuracy of the SHWS. However, the primary concern with rolling shutter mode is the potential for image distortion affecting wavefront aberration measurements.

Using the parameters of our Hamamatsu Lightning camera, we consider this concern and find that image distortion caused by the rolling shutter is largely inconsequential for AO ophthalmoscopy. For our rolling shutter camera, the difference in exposure start time between the top and the bottom rows of pixels in our region of interest (1920 W × 1840 H pixels) is 2.9 ms, limiting the maximum frame rate of the camera to 342 Hz. Hence, for aberrations that change more slowly than 2.9 ms (or 342 Hz), image distortion is minimal and will not impact aberration measurements. Aberrations that change faster than 2.9 ms will experience some distortion, but these aberrations are exceedingly small. Based on our measurements of ocular aberration power spectra (Supplementary Figure 2) and the assumption that ocular aberrations follow a power law, the portion of the aberrations changing faster than 2.9 ms (or 342 Hz) is <0.1% of the total aberration power.

The requirement for correcting aberrations is actually less stringent than the requirement for measuring them (discussed in the last paragraph). This is because the goal of AO is to minimize the displacements of the focal spots formed by the SHWS lenslets (sub-apertures). Therefore, minimizing the displacement of a single focal spot depends only on those rows of pixels associated with that specific lenslet. Our SHWS has 20 rows of lenslets, so the distortion for each focal spot image is minimal for aberrations that change more slowly than ~2.9 ms/20 = 0.145 ms (or 6.9 kHz). Based on our measurements of the ocular aberration power spectra (Supplementary Figure 2) and the assumption that ocular aberrations follow a power law, we expect that aberrations changing faster than 0.145 ms (or 6.9 kHz) contribute extremely little (<0.01% of the total power). Even if such aberrations were significant and caused distortion with rolling shutter, their frequency (6.9 kHz) is so much higher than the bandwidth of our AO system (38 Hz, the maximal frequency our AO system can correct). Thus, the AO system would not correct these aberrations, whether distorted or not.

This analysis, based on the specific parameters of our wavefront sensor camera, can also be applied to other cameras.

Supplementary Note 2: AO system modeling and the relationship between the power rejection magnitude and the product of AO loop rate and loop gain.

We modeled our ultrafast AO system as a linear cascade of transfer functions as depicted in Supplementary Figure 4, and then used this model to analyze the temporal and noise performance of our AO. The model is general and can be applied to analyze other closed-loop AO systems.

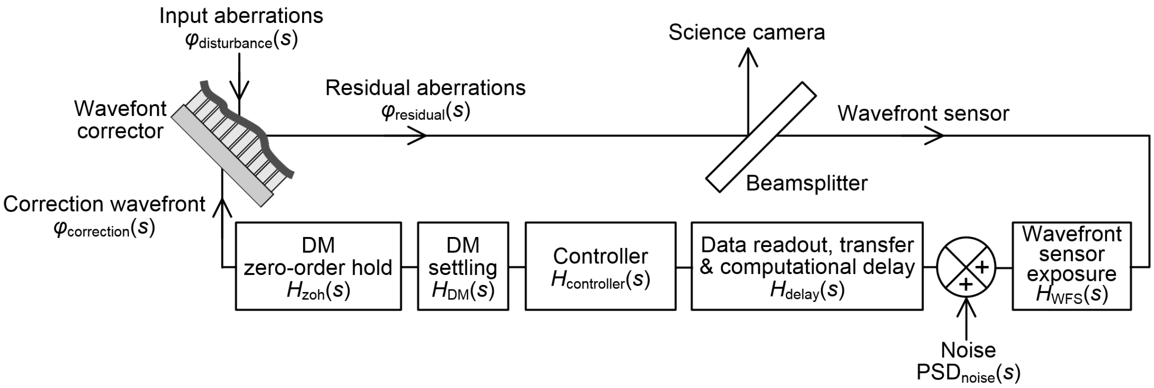

Supplementary Figure 4. Block diagram of a general closed-loop AO system for modeling its temporal and noise performance. $\varphi_{\text{disturbance}}(s)$ represents the aberrations injected into the AO system, $\varphi_{\text{correction}}(s)$ represents the correction applied to the DM, and $\varphi_{\text{residual}}(s)$ represents the residual aberrations after the applied correction. The AO open-loop transfer function $H_{\text{OL}}(s)$ is a linear cascade of five transfer functions, $H_{\text{OL}}(s)$ = $H_{\text{WFS}}(s)\times H_{\text{delay}}(s)\times H_{\text{controller}}(s)\times H_{\text{DM}}(s)\times H_{\text{zoh}}(s)$, each defined in the text. DM is the deformable mirror.

Expressions for the five transfer functions in Supplementary Figure 4 are introduced below [5-8].

$$H_{\text{WFS}}(s) = \frac{1-\exp(-sT_{\text{integration}})}{sT_{\text{integration}}},$$

where $T_{\text{integration}}$ is the integration time (or exposure time) of the SHWS camera, $s = i2\pi f = i\omega$, $i^2 = -1$, f is the temporal frequency, and $\omega = 2\pi f$ is the angular frequency.

$$H_{\text{delay}}(s) = \exp(-sT_{\text{delay}}),$$

where $T_{\text{delay}} = T_{\text{readout\&transfer}} + T_{\text{computation}}$, $T_{\text{readout\&transfer}}$ is the time delay due to sensor readout and data transfer from camera to computer, and $T_{\text{computation}}$ is the time duration needed to process the data on the computer.

$$H_{\text{controller}}(s) = \frac{\textit{loop gain}}{1-\exp(-sT_{\text{hold}})}$$

models the integrator controller, where T_{hold} = 1/(AO loop rate) and is the time duration for which the DM holds its shape; *loop gain* is the integral gain of the integral controller.

$$H_{\text{zoh}}(s) = \frac{1-\exp(-sT_{\text{hold}})}{sT_{\text{hold}}}$$

models the zero-order hold of the DM.

$$H_{\text{DM}}(s) = \frac{1}{1+T_{\text{DM}}\, s/(2\pi i)}$$

models the DM as a low-pass filter. T_{DM} is the time it takes for the DM to stabilize after actuation and is modeled by the time response of a single DM actuator as

$$D(t) = D_{\text{final}}\left[1 - \exp(-t/T_{\text{DM}})\right],$$

where $D(t)$ is the displacement of the DM actuator at time t, and D_{final} is the final displacement when the DM actuator reaches its stable state. As expressed, T_{DM} is a time constant depicting when the DM displacement reaches 63.2% of its final value. From the manufacturer's specification sheet (see Supplementary Figure 5) of our DM (DM97-15 high speed, ALPAO; purchased in 2010), $T_{DM} \approx 0.55$ ms, which is used in our theoretical and numerical calculations. To the best of our knowledge, prior ophthalmic AO analyses in the literature have neglected the transient DM response, assuming it to be fast enough in relation to the AO system speed to be inconsequential. However for ultrafast AO, this assumption may not hold true, and thus, we have included its impact on AO performance.

The rejection transfer function, $H_{\text{reject}}(s) = \varphi_{\text{residual}}(s)/\varphi_{\text{disturbance}}(s)$, quantifies the capability of an AO system to reduce input aberrations as a function of temporal frequency and is expressed in terms of the open-loop transfer function [9], $H_{OL}(s)$, as

$$H_{\text{reject}}(s) = 1/[1+ H_{OL}(s)], \tag{S1}$$

where $H_{OL}(s) = H_{WFS}(s)\times H_{\text{delay}}(s)\times H_{\text{controller}}(s)\times H_{DM}(s)\times H_{\text{zoh}}(s)$. (S2)

The power rejection magnitude, defined as $|H_{\text{reject}}(s)|^2$, can thus be calculated by

$$\begin{aligned}|H_{\text{reject}}(s)|^2 &= |1/[1+ H_{OL}(s)]|^2\\ &= |1/[1+H_{WFS}(s)H_{\text{delay}}(s)H_{\text{controller}}(s)H_{DM}(s)H_{\text{zoh}}(s)]|^2\\ &= \frac{1}{\left|1+\dfrac{1-\exp(-sT_{\text{integration}})}{sT_{\text{integration}}}\dfrac{\exp(-sT_{\text{delay}})}{s}\dfrac{1}{1+T_{DM}\,s/(2\pi i)}\times\left(loop\ rate\times loop\ gain\right)\right|^2},\end{aligned} \tag{S3}$$

revealing that the power rejection magnitude depends on the product of the AO loop rate and loop gain and three system time constants. Equation (S3) is presented as Equation (1) in the main text. The Matlab code used to calculate the power rejection curve based on Equation (S3) is provided as Supplementary Software.

Supplementary Note 3: Relationship between the AO bandwidth and the product of AO loop rate and loop gain.

The bandwidth of an AO system is equal to the cutoff frequency (also called the crossover frequency) of the power rejection curve and is defined as the frequency at which the power rejection magnitude first reaches 1 [see Figure 1e, f] [5, 7, 10]. Therefore, to determine the AO bandwidth, we let $|H_{\text{reject}}(s)|^2 = 1$, which is equivalent to

$$|1/[1+ H_{OL}(s)]|^2 = 1. \tag{S4}$$

We can express the open-loop transfer function in polar coordinates as

$$H_{OL}(s) = A(s)\exp[j\varphi(s)],$$

where $A(s)$ and $\varphi(s)$ are the amplitude and phase parts of the transfer function, respectively. By inserting $H_{OL}(s) = A(s)\exp[j\varphi(s)]$ into Equation (S4), we get

$$|1/\{1+ A(s)\exp[j\varphi(s)]\}|^2 = 1,$$

with the non-trivial solution being

$$A(s) = -2\cos[\varphi(s)]. \tag{S5}$$

From Equation (S2), we know

$$\begin{aligned}H_{OL}(s) &= H_{WFS}(s)\times H_{\text{delay}}(s)\times H_{\text{controller}}(s)\times H_{DM}(s)\times H_{\text{zoh}}(s)\\ &= \frac{1-\exp(-sT_{\text{integration}})}{sT_{\text{integration}}}\frac{\exp(-sT_{\text{delay}})}{s}\frac{1}{1+T_{DM}\,s/(2\pi i)}\times\left(loop\ rate\times loop\ gain\right).\end{aligned}$$

By inserting $s = i\omega$ into the above expression for $H_{\mathrm{OL}}(s)$ and rearranging the terms, we obtain the expressions for its amplitude and phase parts. That is

$$H_{\mathrm{OL}}(\omega) = (loop\ rate \times loop\ gain) \times \frac{1-\exp(-i\omega T_{\mathrm{integration}})}{i\omega T_{\mathrm{integration}}} \frac{\exp(-i\omega T_{\mathrm{delay}})}{i\omega} \frac{1}{1+T_{\mathrm{DM}}\omega/2\pi}$$

$$= \frac{2(loop\ rate \times loop\ gain)}{-\omega^2 T_{\mathrm{integration}}} \times \sin\left(\frac{\omega T_{\mathrm{integration}}}{2}\right) \times \frac{1}{1+T_{\mathrm{DM}}\omega/2\pi} \times \exp\left\{ i \arctan\left\{ \cot\left[\frac{\omega(2T_{\mathrm{delay}}+T_{\mathrm{integration}})}{2}\right]\right\}\right\} \quad \text{(S6)}$$

and therefore

$$A(\omega) = \frac{2(loop\ rate \times loop\ gain)}{-\omega^2 T_{\mathrm{integration}}} \times \sin\left(\frac{\omega T_{\mathrm{integration}}}{2}\right) \times \frac{1}{1+T_{\mathrm{DM}}\omega/2\pi}, \quad \text{(S7)}$$

$$\varphi(\omega) = \arctan\left\{ \cot\left[\frac{\omega(2T_{\mathrm{delay}}+T_{\mathrm{integration}})}{2}\right]\right\}. \quad \text{(S8)}$$

By inserting Eqs. (S7) and (S8) into Equation (S5), we get

$$\frac{2(loop\ rate \times loop\ gain)}{-\omega^2 T_{\mathrm{integration}}} \times \sin\left(\frac{\omega T_{\mathrm{integration}}}{2}\right) \times \frac{1}{1+T_{\mathrm{DM}}\omega/2\pi} = -2\cos\left\{ \arctan\left\{ \cot\left[\frac{\omega(2T_{\mathrm{delay}}+T_{\mathrm{integration}})}{2}\right]\right\}\right\},$$

which after rearranging is equivalent to

$$T_{\mathrm{integration}} \sin\left[\frac{\omega(2T_{\mathrm{delay}}+T_{\mathrm{integration}})}{2}\right]\omega^2 (1+T_{\mathrm{DM}}\omega/2\pi) - (loop\ rate \times loop\ gain) \times \sin\left(\frac{\omega T_{\mathrm{integration}}}{2}\right) = 0. \quad \text{(S9)}$$

By inserting $\omega = 2\pi f_{\mathrm{c}}$ into Equation (S9), we obtain the equation for determining the AO bandwidth f_{c}:

$$T_{\mathrm{integration}} (2\pi f_{\mathrm{c}})^2 (1+T_{\mathrm{DM}} f_{\mathrm{c}}) \times \sin[\pi f_{\mathrm{c}}(2T_{\mathrm{delay}}+T_{\mathrm{integration}})] - (loop\ rate \times loop\ gain) \times \sin(\pi f_{\mathrm{c}} T_{\mathrm{integration}}) = 0. \quad \text{(S10)}$$

Similar to Equation (S3) for the power rejection magnitude, Equation (S10) shows that f_{c} depends on the product of the AO loop rate and loop gain and three system time constants. Equation (S10) is presented as Equation (2) in the main text.

Supplementary Note 4: Impact of DM actuation on AO performance and method to minimize this effect while maximizing AO temporal performance.

Ultrafast AO uses a short SHWS integration time to reduce latency. However, when integration times are shorter than or similar to the response time of the DM, the DM actuation can potentially corrupt the SHWS measurement, leading to reduced AO performance. We study this effect here using the ultrafast AO system and develop a way to eliminate it.

Supplementary Figure 5 shows a representative step response of our DM (high-speed DM97-15, ALPAO; purchased in 2010) as provided by the manufacturer. In the figure, the response takes 1 ms to reach 95% of the final stable displacement (hence the rise time = 1 ms), followed by ringing. The ringing becomes unobservable at ~5 ms. If the SHWS camera exposes during DM actuation and if the exposure time is shorter than or comparable to the DM's rise time, the SHWS may measure an aberration that differs from the true aberration after the DM has fully settled. Hence, to mitigate this potential error, the SHWS exposure can be delayed until after the DM settles. We can approximate this delay using the nominal DM rise time given in Supplementary Figure 5 (1 ms). However, for our specific DM, we found greater precision by directly measuring the impact of individual actuators on the SHWS measurement. Using this approach, we iteratively determined the optimal exposure delay that minimizes the effect of the DM actuation and maximizes the AO loop rate. This will be described in detail next.

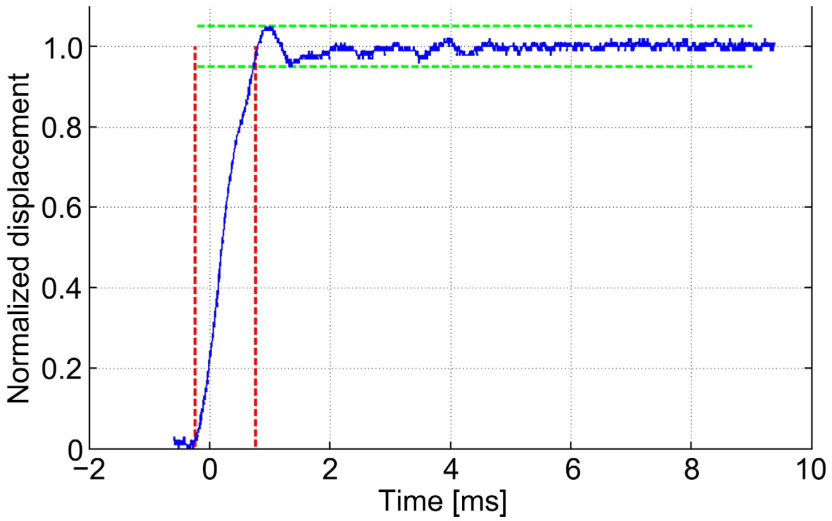

Supplementary Figure 5. Typical step response of our DM as provided by the manufacturer, ALPAO Inc. The rise time (to reach 95% of the final stable displacement) is 1 ms, followed by ringing. According to the manufacturer, the rise time is independent of the actuator stroke, making the response curve representative across different stroke sizes.

The discontinuous-exposure scheme allows us to control the SHWS camera exposure start time relative to when DM actuation begins (Supplementary Figure 1). To study the DM actuation effect, we controlled the delay between the control commands sent to the DM and the start of the SHWS exposure (i.e. the exposure delay shown in Supplementary Figure 1). This was realized by using the "external trigger delay function" of the SHWS camera. The function is executed by the onboard microcontroller of the camera, so timing accuracy (10 µs) and repeatability is much better than that of the software delay function provided by the Windows™ operating system. Our SHWS camera uses a rolling shutter that sweeps from top to bottom of the sensor, with four adjacent rows of the sensor exposed and read out simultaneously. Thus in this mode, every four rows begin exposing at slightly different times (separated by 6.3 µs); the top four rows are exposed first and the bottom four rows last. For this timing study, the exposure time for each row of the camera sensor was set at 0.1 ms, which is comparable to what we used for the subject imaging and is much shorter than the nominal DM rise time of 1 ms. The AO loop gain was 1.

The step responses to a static aberration (−2 D cylinder, axis along the horizontal direction) for exposure delays of 0, 0.3, 1 and 6 ms are shown in Supplementary Figure 6. A 0-ms delay exposes the SHWS to almost the entire DM response and a 6-ms delay avoids it entirely, a 1-ms delay is equal to the nominal rise time of the DM, and as discussed below a 0.3-ms delay is the optimum delay for minimizing the DM actuation effect while maximizing AO loop rate and bandwidth.

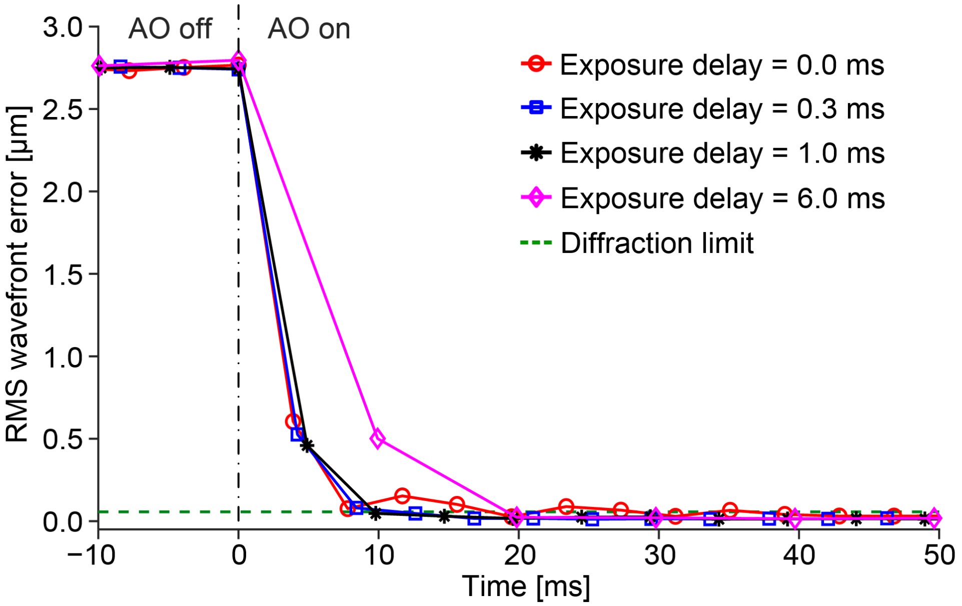

Supplementary Figure 6. RMS wavefront error as a function of time before and after AO onset (time 0) in the presence of a static aberration (−2 D cylinder added on the DM) for different exposure delays.

As evident in Supplementary Figure 6, we observe overshoot of the step response to the aberration with the 0-ms exposure delay, whereas there was no overshoot with the 0.3-, 1- and 6-ms delays. The SHWS lenslet spot displacements in Frames #3-#9 (Supplementary Figure 7) are strong evidence of degradation due to DM actuation. The axis of the aberration (cylinder) was along the horizontal direction, so the spot displacements were mainly vertical and hence we plot only the signed vertical spot displacement in Supplementary Figure 7. The rows of lenslets affected by DM actuation were determined by comparing the lenslet spot displacements when the exposure delay was 0 ms to those when the exposure delay was 6 ms, at which point the DM ringing depicted in Supplementary Figure 5 has completely subsided. With an exposure delay of 0 ms (red dots in Supplementary Figure 7), the spots in the top four rows oscillated between negative and positive displacements. The oscillations of the top two rows had a much larger amplitude, with their displacements remaining quite large even in the 9th frame after closing the AO loop; this caused the overshoot in the RMS wavefront error evident in Supplementary Figure 6. By contrast, the spot displacements for the 0.3-, 1-, and 6-ms delays were much smaller and remained fairly stable after Frame #2. Because the rolling shutter of the SHWS sweeps from top to bottom, the 1st row of lenslets was affected more by DM actuation than the 2nd row. The displacements of the top two rows for 0-ms delay in the 1st frame after AO onset (Frame #1) are similar to those before AO activation (Frame #0), showing that these two rows of the SHWS barely see the first DM correction after AO activation; in other words, DM actuators had just started moving and had not reached their final positions when the top two rows were exposed. Thus, the wavefront sensor does not accurately measure the corrected aberration because it sees only a fraction of the correction in the top rows. These partial-correction measurements cause the system to over-correct because in each frame it sees residual aberrations that have actually been corrected, leading to an oscillatory behavior.

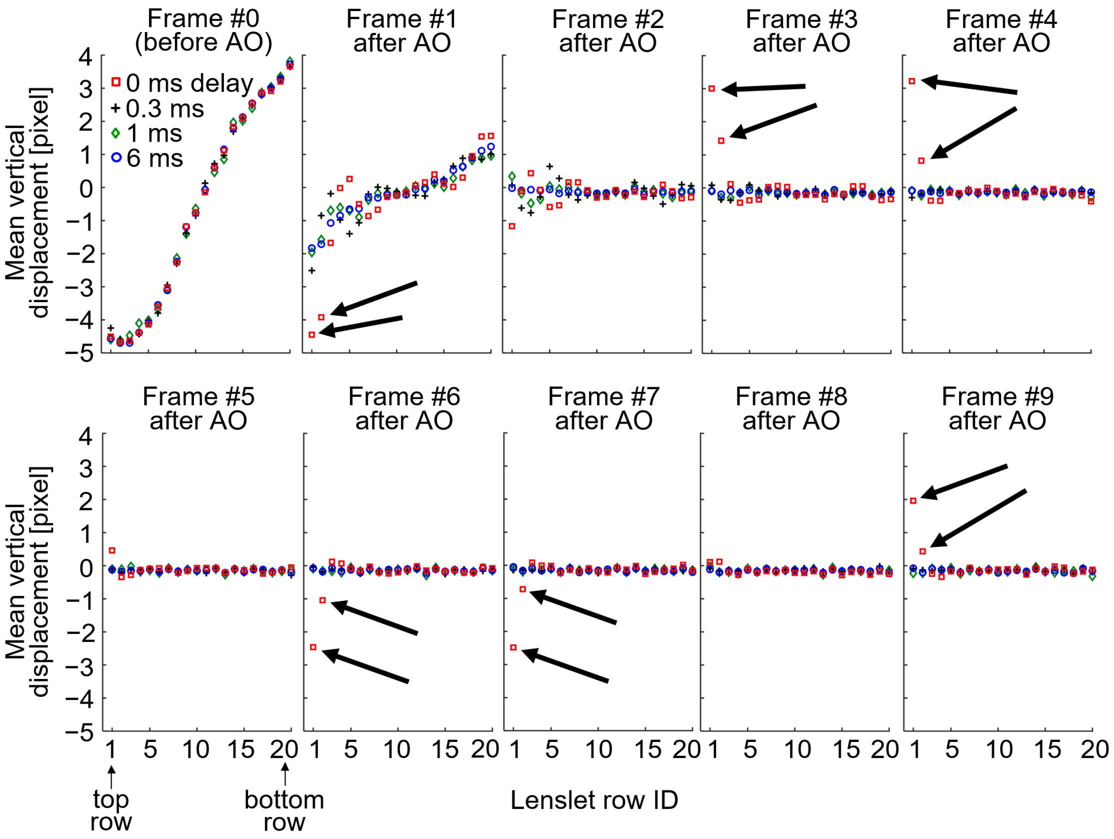

Supplementary Figure 7. DM actuation effect is revealed by the mean vertical displacement of the lenslet spots in various rows of the microlens array. Our microlens array has 20 × 20 square lenslets. Row #1 to Row #20 on the lenslet array corresponds to top to bottom on the SHWS camera, and the rolling shutter of the camera sweeps from top to bottom. Frame #0 is the last frame before AO is turned on, and Frame #i is the i-th frame after AO is turned on. A positive vertical displacement means the centroid of the lenslet spot is above the reference spot taken when a flat wavefront illuminated the SHWS, while a negative vertical displacement means the centroid is below the reference spot. The input aberration was a −2 D cylinder with an axis along the horizontal direction and applied to the DM. DM actuation effect is revealed by the large spot displacements of the top two rows (denoted by the arrowheads), which occur even in the 9th frame after AO onset with an exposure delay of 0 ms. The spot displacements of the top two rows oscillate over time between negative and positive values, corresponding to the overshoot of RMS wavefront error seen in Supplementary Figure 6 when the exposure delay is 0 ms.

Because we found that DM actuation mainly affected the top two rows of SHWS lenslets (out of 20) and the rolling shutter exposes rows from top to bottom in 2.9 ms, we determined the optimal exposure delay to be 2.9 × 2/20 = 0.29 ms ≈ 0.3 ms. This optimal delay allows us to achieve a higher AO loop rate than that achieved using the nominal DM rise time (1 ms, from Supplementary Figure 5) while avoiding the effects of DM actuation. Indeed, when we used this optimal delay of 0.3 ms, no overshoot occurred in the step response to the aberration (−2.0 D cylinder, Supplementary Figure 6) and the AO loop rate increased by 17% to 233 Hz compared with the loop rate (201 Hz) achieved using an exposure delay of 1 ms, the nominal DM rise time. Similarly, the AO bandwidth increased 17% from 32.5 Hz to 38.0 Hz when the exposure delay was reduced from 1 ms to 0.3 ms.

In summary, DM actuation can degrade AO performance when the exposure time is shorter or comparable to the DM rise time. We introduce a method to determine the optimal exposure delay (0.3 ms for our system) under discontinuous-exposure scheme, effectively eliminating AO overshoot while maximizing the AO bandwidth. By contrast, because the continuous-exposure scheme inherently uses a 0-ms exposure delay, its

AO performance would be degraded by DM actuation when the exposure time is shorter or comparable to the DM rise time. This is especially relevant for high-speed AO systems.

Supplementary Note 5: Determining the noise performance of our ultrafast AO system in terms of its residual wavefront error and noise transfer function

Aberration correction by an AO system is not perfect, leaving residual uncorrected wavefront error. There are three main sources of residual wavefront error σ_{residual} [11, 12]: (1) temporal error σ_{temporal} due to the time lag between wavefront sensing measurement and correction; (2) wavefront sensing error σ_{noise} due to noise accumulated in the sensor image and its propagation through the AO loop; and (3) fitting error σ_{fitting} due to insufficient number of lenslets or dynamic range to perfectly sample the aberrations or insufficient number of DM actuators or stroke to perfectly correct the aberrations. The total residual wavefront error is the square root of the sum of these three errors, i.e. $\sigma_{\text{residual}} = \sqrt{\sigma^2_{\text{temporal}} + \sigma^2_{\text{noise}} + \sigma^2_{\text{fitting}}}$. Because our AO samples the eye pupil with 300 lenslets and 97 high-stroke DM actuators (resulting in a likely small σ_{fitting} in most cases) and because σ_{fitting} is a spatial rather than temporal or noise property of the AO, we ignore σ_{fitting} in the following analyses. This section focuses on determining the residual wavefront error attributed to σ_{noise}, and next section focuses on determining the residual wavefront error attributed to σ_{temporal}.

Wavefront sensing noise is composed of photon shot noise and camera noise (typically dominated by readout noise) that cause centroiding errors in the SHWS spots. These noises can be estimated from the acquired SHWS images. For the thresholding center of gravity method that we use (described in Methods), the centroiding error in terms of phase difference across a lenslet due to photon shot noise $\sigma_{\varphi,\text{ photon}}$ and camera readout noise $\sigma_{\varphi,\text{ readout}}$ can be determined by

$$\sigma_{\varphi,\text{ photon}} = \sqrt{\frac{\pi^2}{2\ln 2}\frac{1}{N_{\text{ph}}}\left(\frac{N_{\text{T}}}{N_{\text{samp}}}\right)^2}, \quad \text{(M1)}$$

$$\sigma_{\varphi,\text{ readout}} = \sqrt{\frac{\pi^2}{3}\frac{N^2_{\text{readout}}}{N^2_{\text{ph}}}\frac{N^4_{\text{S}}}{N^2_{\text{samp}}}}, \quad \text{(M2)}$$

where N_{ph} is the number of photoelectrons per lenslet per frame after thresholding; $N_{\text{samp}} = 2.1$ in our case is the diffraction-limited Airy disk size in pixels sampled by our wavefront sensor; N_{T} is the full-width half-maximum of the experimental focal spot size; $N_{\text{readout}} = 2e^-$ is the readout noise of our wavefront sensor; and N^2_{S} is the number of pixels used for centroid calculation after thresholding [7, 13]. The total wavefront sensing error is calculated by $\sigma_{\varphi,\text{ total}} = \sqrt{\sigma^2_{\varphi,\text{ photon}} + \sigma^2_{\varphi,\text{ readout}}}$.

To study the impact of the wavefront sensing noise on the residual wavefront error $\varphi_{\text{residual}}(s)$ (i.e., as seen by the retinal camera (science camera) in Supplementary Figure 4), we need to know how the wavefront sensing error propagates through the AO loop. This error propagation is quantified by the noise transfer function [7, 8, 14, 15]. From Supplementary Figure 4,

$$\begin{aligned} H_{\text{noise}}(f) &= \frac{\varphi_{\text{residual}}(f)}{\text{PSD}_{\text{noise}}(f)} \\ &= -H_{\text{reject}}(f)\frac{H_{\text{OL}}(f)}{H_{\text{WFS}}(f)}, \end{aligned} \quad \text{(M3)}$$

where transfer functions $H_{\text{reject}}(f)$, $H_{\text{OL}}(f)$, and $H_{\text{WFS}}(f)$ are defined in Supplementary Note 2. As the AO loop gain and loop rate affect $H_{\text{OL}}(f)$ and $H_{\text{reject}}(f)$, they affect the noise transfer function. The Matlab code used to calculate the noise transfer function based on Equation (M3) is provided as Supplementary Software.

Once we know the noise transfer function H_{noise} and the total wavefront sensing error $\sigma_{\varphi,\,\text{total}}$, the RMS wavefront error due to wavefront sensing noise can be found by [7, 8, 14, 15]

$$\sigma_{\text{noise}} = \sqrt{\int_0^{f_s/2} \text{PSD}_{\text{noise}}(f) \times \left|H_{\text{noise}}(f)\right|^2 df}\ , \tag{M4}$$

where $\text{PSD}_{\text{noise}}(f)$ is the power spectrum density (PSD) of wavefront sensing noise; f_s is the sampling rate of the wavefront sensor. Because the wavefront sensing noise is white noise, $\text{PSD}_{\text{noise}}(f)$ is a flat line with $\int_0^{f_s/2} \text{PSD}_{\text{noise}}(f) df = \sigma^2_{\varphi,\,\text{total}}$.

Supplementary Note 6: Predicting the required temporal performance of an AO system in terms of its residual wavefront error

To predict how fast an AO system must operate for a particular clinically-relevant scenario, we compute the temporal error σ_{temporal} as a function of AO loop rate. We then find the minimal AO loop rate that achieves diffraction-limited performance (i.e. when σ_{temporal} is equal to $\lambda/14$ and λ is the wavelength).

Once we measure the aberration dynamics from an eye (characterized by its PSD spectrum $\text{PSD}_{\text{eye}}(f)$), the RMS wavefront error due to temporal error can be calculated by [5, 7, 8, 14, 15]

$$\sigma_{\text{temporal}} = \sqrt{\int_0^{f_s/2} \text{PSD}_{\text{with AO}}(f) df} = \sqrt{\int_0^{f_s/2} \text{PSD}_{\text{eye}}(f) \times \left|H_{\text{reject}}(f)\right|^2 df}\,, \tag{M5}$$

where f_s is the camera frame rate (= 342 Hz), $\left|H_{\text{reject}}(f)\right|^2$ is the power rejection curve, and $H_{\text{reject}}(f)$ is the rejection transfer function (See Supplementary Note 2). In practice, the measured power spectrum $\text{PSD}_{\text{eye}}(f)$ decreases with increasing temporal frequency (following a power law [12, 16]) until it reaches a plateau determined by the noise floor (See Supplementary Figure 2). We use f_n to denote the frequency at which $\text{PSD}_{\text{eye}}(f)$ first deviates from the power law. To estimate the $\text{PSD}_{\text{eye}}(f)$ values in the frequency range between f_n and $f_s/2$ where most signal is below the noise floor, we use curve fitting and extrapolation. First, we fit the measured $\text{PSD}_{\text{eye}}(f)$ data between 1 Hz and f_n using a power law model $y(f) = a \times f^b$, where a and b are fitting parameters, and then extrapolate the $\text{PSD}_{\text{eye}}(f)$ values between f_n and $f_s/2$ by using the fitted power law model.

Supplementary Note 7: Effect of the neutral density filters on the wavefront measurement is negligible.

Because the mimicked conventional AO used a much longer integration time (45 ms) than ultrafast AO (0.126 ms), we employed neutral density (ND) filters before the wavefront sensor to ensure that the photon count on the SHWS accumulated during a 45 ms exposure matched that accumulated during 0.126 ms exposure for ultrafast AO. This ensured the same signal-to-noise ratio for both conditions and avoided saturating the SHWS camera. Here, we present experimental results and show the effect of the ND filters on the wavefront measurement is negligible.

We mounted a model eye (composed of an achromatic lens and a business card as the retina) in the system and measured its aberration with and without using the ND filters. The exposure time was 45 ms with the ND filters and 0.126 ms without the ND filters, respectively. The wavefront measured with and without the ND filters are shown in Supplementary Figure 8a and 8b, and their difference is shown in Supplementary Figure 8c and 8d (with different scales).

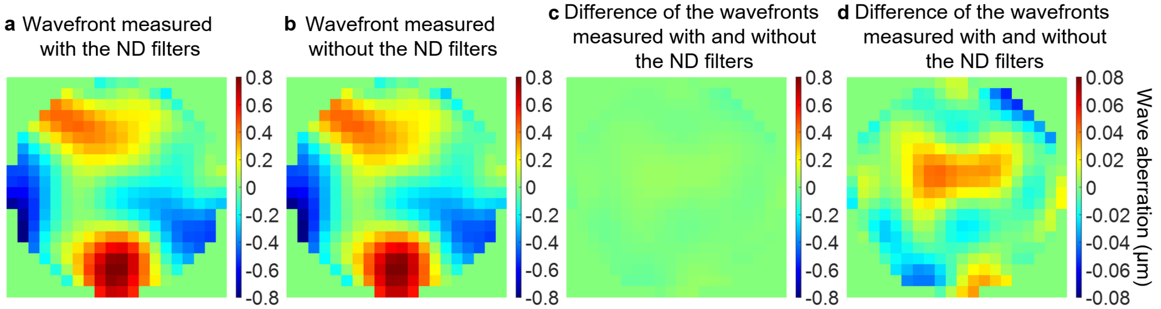

Supplementary Figure 8. Effect of ND filters on the wavefront measurement. **a** Wavefront measured with the ND filters. **b** Wavefront measured without the ND filters. **c**, **d** Difference of the wavefronts measured with and without the ND filters, plotted in two different scales. The experiment was repeated six times with similar results.

The difference of the two wavefronts is very small, with an RMS of 0.018 µm, much smaller than the diffraction limit of 0.056 µm (the Maréchal criterion). We repeated this measurement 6 times. The mean RMS of the differential wavefront was 0.022 µm, again well below the diffraction limit of 0.056 µm. The RMS of the wavefront measured with and without the ND filters are shown in Supplementary Figure 9. The RMS of the wavefront measured with the ND filters was 0.309 ± 0.004 µm (Mean ± SD, n = 6) and the RMS of the wavefront measured without the ND filters was 0.311 ± 0.003 µm (Mean ± SD, n = 6). Two-sample t-test does not reject the null hypothesis that the mean of the RMS measured with and without ND filters are the same ($p = 0.39$), at the 5% significance level even if equal variances are not assumed. Based on these results, we conclude that the ND filters have a negligible effect on the measured wavefront.

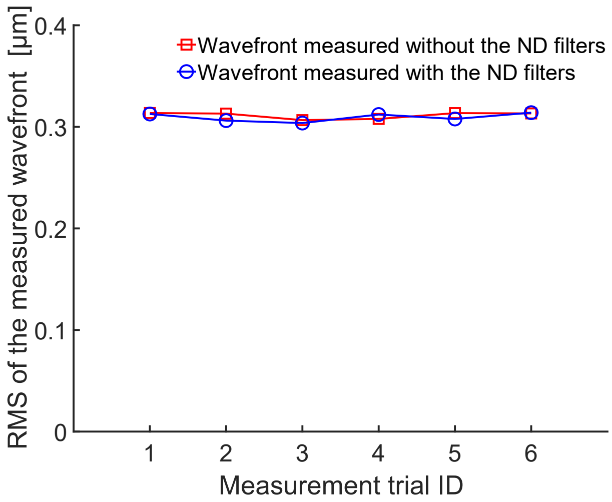

Supplementary Figure 9. RMS of the wavefront measured with and without the ND filters for six repeated measurements.

Supplementary Note 8: Matlab code for generating the pseudo-random aberrations (pink noise) that are applied to the DM, in order to measure the power rejection curve.

The pseudo-random aberrations (P[n]) that we applied to the DM consisted of pink noise, whose PSD follows $1/f$ [17]. Because we used an integral controller and a direct slope reconstruction method [5, 18, 19], the voltages (V) that were applied to the DM's 97 actuators followed

$V[n] = V[n-1]$, when AO correction was not applied, and

$V[n] = V[n-1] - g \times C \times D[n]$, when AO correction was applied,

where g is the AO loop gain, C is the control matrix that relates DM actuator responses to SHWS lenslet spot displacements, and D is a vector of lenslet spot displacements measured at uniformly spaced time points, n = 1, 2, …

In order for the DM to generate P[n], which is subsequently measured by the SHWS, the difference dP[n] = P[n] – P[n–1] is applied to the DM at each time point n, so that when

(1) AO correction is not applied: $V[0] = P[0]$; $V[n] = V[n-1] + dP[n] = P[n]$, n = 1, 2, …, and

(2) AO correction is applied: $V[0] = P[0]$; $V[n] = V[n-1] - g \times C \times D[n] + dP[n]$, n = 1, 2, …

The Matlab code to generate dP[n] is shown below and provided as Supplementary Software.

```
N = 1000; % # of time points to be generated.
N = N+1;
num_actuators = 97; % # of actuators on the DM.
y = pinknoise(N,num_actuators); % Generate N time points of pink noise for each actuator.
k=0.2;
for ii=1:num_actuators
   y1 = y(:,ii);
   max_y = max(max(y1),-min(y1));
   y1_norm = y1/max_y*k;
   y_norm(:,ii) = y1_norm;
end
y_norm_diff = diff(y_norm);
y_norm_diff(1,:) = y_norm_diff(1,:)+y_norm(1,:); % The pseudo-random aberrations (pink noise) to be injected to the DM.
```

References

1. D. Wang, E. H. Zhou, J. Brake, H. Ruan, M. Jang, and C. Yang, "Focusing through dynamic tissue with millisecond digital optical phase conjugation," Optica 2, 728-735 (2015).

2. Y. Liu, C. Ma, Y. Shen, J. Shi, and L. V. Wang, "Focusing light inside dynamic scattering media with millisecond digital optical phase conjugation," Optica 4, 280-288 (2017).

3. Andor, "Zyla sCMOS user guide," (2022).

4. PCO, "PCO sCMOS Cameras E-Book," (2024).

5. D. T. Miller and A. Roorda, "Adaptive optics in retinal microscopy and vision," Handbook of optics 3, 15.11-15.30 (2009).

6. H. Hofer, L. Chen, G. Y. Yoon, B. Singer, Y. Yamauchi, and D. R. Williams, "Improvement in retinal image quality with dynamic correction of the eye's aberrations," Opt. Express 8, 631-643 (2001).

7. F. Roddier, Adaptive Optics in Astronomy (Cambridge University Press, Cambridge, 1999).

8. C. Correia, Advanced control laws for the new generation of AO systems, Proc. of SPIE Vol. 10703, 107031M (2018).

9. N. S. Nise, Control systems engineering (John Wiley & Sons, 2020).

10. K. M. Hampson, R. Turcotte, D. T. Miller, K. Kurokawa, J. R. Males, N. Ji, and M. J. Booth, "Adaptive optics for high-resolution imaging," Nature Reviews Methods Primers 1, 68 (2021).

11. J. W. Hardy, Adaptive optics for astronomical telescopes (Oxford University Press on Demand, 1998), Vol. 16.

12. J. Jarosz, P. Mecê, J.-M. Conan, C. Petit, M. Paques, and S. Meimon, "High temporal resolution aberrometry in a 50-eye population and implications for adaptive optics error budget," Biomed. Opt. Express 8, 2088-2105 (2017).

13. S. Thomas, T. Fusco, A. Tokovinin, M. Nicolle, V. Michau, and G. Rousset, "Comparison of centroid computation algorithms in a Shack–Hartmann sensor," Mon. Not. R. Astron. Soc. 371, 323-336 (2006).

14. S. Meimon, C. Petit, T. Fusco, and C. Kulcsar, "Tip–tilt disturbance model identification for Kalman-based control scheme: application to XAO and ELT systems," J. Opt. Soc. Am. A 27, A122-A132 (2010).

15. L. A. Poyneer, D. W. Palmer, B. Macintosh, D. Savransky, N. Sadakuni, S. Thomas, J.-P. Véran, K. B. Follette, A. Z. Greenbaum, S. Mark Ammons, V. P. Bailey, B. Bauman, A. Cardwell, D. Dillon, D. Gavel, M. Hartung, P. Hibon, M. D. Perrin, F. T. Rantakyrö, A. Sivaramakrishnan, and J. J. Wang, "Performance of the Gemini Planet Imager's adaptive optics system," Appl. Opt. 55, 323-340 (2016).

16. H. Hofer, P. Artal, B. Singer, J. L. Aragón, and D. R. Williams, "Dynamics of the eye's wave aberration," J. Opt. Soc. Am. A 18, 497-506 (2001).

17. E. Gofas-Salas, P. Mecê, C. Petit, J. Jarosz, L. M. Mugnier, A. Montmerle Bonnefois, K. Grieve, J. Sahel, M. Paques, and S. Meimon, "High loop rate adaptive optics flood illumination ophthalmoscope with structured illumination capability," Appl. Opt. 57, 5635-5642 (2018).

18. W. Jiang and H. Li, Hartmann-Shack wavefront sensing and wavefront control algorithm, Proc. SPIE Vol. 1271, 82-93 (1990).

19. C. Boyer, V. Michau, and G. Rousset, Adaptive optics: interaction matrix measurements and real time control algorithms for the COME-ON project, Proc. SPIE 1237, Vol. 1271, 406-421 (1990).
