# Supplementary material for: Ultrafast adaptive optics for imaging the living human eye
Source: Nat Commun. 2024 Nov 29;15:10409. doi: 10.1038/s41467-024-54687-z (PMC11607088; doi:10.1038/s41467-024-54687-z)
Supplement: Supplementary file 2 — Description of Additional Supplementary Files [file 41467_2024_54687_MOESM2_ESM.pdf]

Description of Additional Supplementary Files

Supplementary Movie 1. Ultrafast AO provides better focus stability in non-cyclopleged eyes. The image was acquired at 2.4° temporal to the fovea. Each image is a projection of a volume along the fast-scan axis, and axial displacement of the retina between fast B-scans was corrected by aligning the photoreceptor layers.

Supplementary Movie 2. Ultrafast AO provides finer focus control in non-cyclopleged eyes.

Supplementary Movie 3. Video of a keratoconic eye shows movement of the contact lens on the cornea after a blink.

Supplementary Movie 4. Video of a nystagmic eye shows involuntary, repetitive movements while viewing the fixation target of the AO-OCT system.

Supplementary Movie 5. **Ultrafast AO enables better-quality cone mosaic images in the nystagmic eye.** Videos were acquired over a 5 s duration. The time points when the DM shape was updated are evident in some conventional AO frames by the horizontal lines, which were generated by an abrupt change in image quality. The AO-OCT volume acquisition rate was 6.7 Hz for a field of view of 1.3° H × 1° V. Because the AO loop rate for conventional AO was only slightly higher (10 Hz), the horizontal line changed positions from frame to frame.
